# Prevalence and factors associated with depression symptoms among school-going adolescents in Central Uganda

**DOI:** 10.1186/s13034-016-0133-4

**Published:** 2016-10-26

**Authors:** Joyce Nalugya-Sserunjogi, Godfrey Zari Rukundo, Emilio Ovuga, Steven M. Kiwuwa, Seggane Musisi, Etheldreda Nakimuli-Mpungu

**Affiliations:** 1 Department of Psychiatry, Makerere University, College of Health Sciences, Kampala, Uganda; 2 Mulago National Referral and Teaching Hospital, Ministry of Health, Kampala, Uganda; 3 Department of Psychiatry, Mbarara University of Science and Technology, Mbarara, Uganda; 4 Department of Psychiatry, Gulu University, Gulu, Uganda; 5 Department of Child Health and Development, Makerere University, College of Health Sciences, Kampala, Uganda

**Keywords:** Depression, Depression symptoms, Adolescents, Orphan-hood, Secondary schools, Uganda

## Abstract

**Background:**

Depression in adolescents constitutes a global public health concern. However, data on its prevalence and associated factors are limited in low income countries like Uganda.

**Methods:**

Using a cross-sectional descriptive study design, 519 adolescent students in 4 secondary schools in Mukono district, Uganda, were randomly selected after meeting study criteria. The 4 school types were: boarding mixed (boys and girls) school; day mixed school; girls’ only boarding school; and, boys’ only boarding school. The 519 participants filled out standardized questionnaires regarding their socio-demographic characteristics and health history. They were then screened for depression using the Children Depression Inventory (CDI) and those with a cut-off of 19 were administered the Mini International Neuro-Psychiatric Interview for Children and Adolescents 2.0 (MINI-KID), to ascertain the Diagnostic and Statistical Manual of Mental Disorders, 4th Edition (DSM IV) diagnostic types of depression and any co morbidity. Logistic regression analyses were used to assess factors associated with significant depression symptoms (a score of 19 or more on the CDI).

**Results:**

There were 301 (58 %) boys and 218 (42 %) girls with age range 14–16 years and a mean age of 16 years (SD 2.18). Of 519 participants screened with the CDI, 109 (21 %) had significant depression symptoms. Of the 109 participants with significant depression symptoms, only 74 were evaluated with the MINI-KID and of these, 8 (11 %) met criteria for major depression and 6 (8 %) met criteria for dysthymia. Therefore, among participants that were assessed with both the CDI and the MINI-KID (n = 484), the prevalence of depressive disorders was 2.9 %. In this sample, 15 (3.1 %) reported current suicidal ideation. In the logistic regression analyses, significant depression symptoms were associated with single-sex schools, loss of parents and alcohol consumption.

**Limitations:**

This is a cross-sectional study therefore, causal relationships are difficult to establish. Limited resources and the lack of collateral information precluded the assessment of a number of potential factors that could be associated with adolescent depression. The MINI-KID was administered to only 74 out of 109 students who scored ≥19 on the CDI since 35 students could not be traced again due to limited resources at the time.

**Conclusions:**

Significant depression symptoms are prevalent among school-going adolescents and may progress to full-blown depressive disorders. Culturally sensitive psychological interventions to prevent and treat depression among school-going adolescents are urgently needed.

## Background

Adolescence has been described as a period of tremendous emotional upheaval and change [1–4]. The transition from childhood to adulthood involves major physical, psychological, cognitive and social transformations [5–8] which may be stressful to the adolescent. These transformational challenges are often associated with emotional turmoil including depression. Indeed a recent review of the mental health burden among children and adolescents world wide indicate that 10–20 % of them in the general population will suffer from at least one mental disorder in a given year [9]. The commonest of these mental health problems is unipolar depressive disorder which has been reported to be associated with a myriad of complications including impaired academic and social functioning and accounting for 40·5 % of disability adjusted life years (DALYs) caused by mental and substance use disorders [10], risky behaviours [11] as well as increased mortality rates through suicide [12].

Considerable literature points to the high prevalence of depression amongst adolescents [13–15]. School based studies of adolescent depression have reported various mean scores ranging between 2.6 and 3.6 % [16–18]. The variation in rates has been attributed to the great diversity in research instruments and methodologies.

The majority of studies documenting adolescent mental problems such as depression are from developed countries. The few studies conducted in sub Saharan African countries that have documented adolescent depression rates indicate estimates of 15.3–37 % among Egyptian students [19, 20] 6.9–23.8 % among Nigerian student populations [21]. In these studies depression has been associated with female gender, alcohol use, poor family functioning, large family size [21], childhood adversities such as emotional neglect [22] and frequent health services use.

Prior studies in Uganda have focused on mental health problems of adolescents in highly vulnerable and marginalised populations such as war traumatised individuals [23] and persons living with human immune deficiency virus (HIV) infection [24]. Further, studies on mental health issues among secondary school students in Uganda have mostly focused on alcohol and substance use problems. In the present study, we use data from four secondary schools to explore the prevalence of depressive symptoms in school-going adolescents. We sought to answer the following questions: What is the prevalence of depressive symptoms in school-going adolescents aged 13–16 years in central Uganda? And to what extent are socio-demographic factors, alcohol/substance use, chronic physical illness, chronic medication use and orphan hood associated with depressive symptoms in this age range?

## Methods

### Study setting and population

Study participants were school-going adolescents recruited from four secondary schools in Mukono district situated in central Uganda where 88 % of the population is rural consisting of peasants who depend on subsistence agriculture for food and as a source of income. Four secondary schools were chosen using stratified random sampling, so that one school was boarding mixed (boys and girls), one day mixed school, one girls’ only boarding school and one boys’ only boarding school.

Of the four selected schools, 3 were boarding schools and 1 was a day school.

### Study procedure

Study data were collected between October and November 2003. The eligibility criteria required participants to be present on the days of interview, be enrolled for at least one year in the participating school, provide assent and have parental/guardian written informed consent. Parents of adolescents in boarding schools were provided with information about the study on visiting days and asked to sign the consent forms thereafter. Adolescents in the day school were provided with information to take to their parents at home who then signed consent forms if they allowed their child to participate in the study. The first author together with research assistants reviewed the study questionnaires with local mental health staff and teachers to ensure local validity and were pretested. Class teachers were asked to distribute study questionnaires to students who were present in class on a given day and were eligible to participate in the study. All questionnaires were administered in English, the official language used in schools. The questionnaires were anonymous and self-administered during regular school hours and took approximately an hour to complete. The first author together with the research assistants checked each questionnaire for any missing data immediately after completion before the student left the study room. Support services and mechanisms of referral for mental health services were available to all participants. The research protocol was approved by the Makerere University School of Medicine Research Ethics Committee, as well as the Uganda National Council of Science and Technology.

### Study measures

#### Socio-demographic variables

In a socio-demographic questionnaire, participants reported their age, gender, marital status of parents, whether their parents were still alive or not, had a physical illness or not, were using any medications, alcohol, drugs or not.

#### Depression symptoms

Depression symptoms were assessed using the self-administered Children’s Depression Inventory (CDI) which is a comprehensive multi-ratter assessment of depressive symptoms in youth aged 7–17 years [25]. The CDI rates symptoms of depression on five subscales namely; negative mood, interpersonal problems, ineffectiveness, anhedonia and negative self-esteem. It comprises of 27 items rated on a 3-point scale [0 (none) to 2 (distinct symptom)]. Total CDI scores range from 0 to 54 with several recommended clinical cut-off scores (e.g., >13; 13–18; ≥19) to indicate elevated depressive symptoms in youth. In this study, participants who scored 19 points or higher were regarded as having significant depression symptoms. The cut-off point of ≥19 was chosen as this has been found more suitable for community participants, with a sensitivity of 94.7 %, a specificity of 95.6 %, a positive predictive value of 0.90, and a negative predictive value of 0.98 [26, 27].

#### Depressive disorder

Participants with significant depression symptoms were recalled for evaluation using Mini International Neuro-Psychiatric Interview for children and adolescents 2.0 (MINI-KID), to ascertain DSM IV diagnosis of depression and co morbidity. This was done by the first author who is a psychiatrist with special training in child and adolescent psychiatry and mental health. However this assessment was conducted on only 74 (68 %) of 109 students who scored ≥19 on the CDI since 35 (32 %) could not be traced.

The MINI-KID is a diagnostic structured interview that was developed for DSM-IV psychiatric disorders [28]. It is organized in diagnostic sections. Using branching-tree logic, the MINI KID has two screening questions per disorder. Additional symptoms within each disorder section are asked only if the screening questions are positively endorsed. The psychometric properties of the MINI-KID have not been described in Uganda but MINI-KID has been used in several studies [29–32].

A diagnosis of current major depression was made if a study participant positively endorsed five or more questions related to depression symptoms and the one question related to functional impairment over the 4-week period prior to the interview. A diagnosis of dysthymia was made if a study participant positively endorsed depressed or irritable mood for at least one year with two or more symptoms related to depression, had not been without the symptoms for more than 2 months at a time, did not meet criteria for major depressive episode, manic or hypomanic episode, psychotic illness, and the symptoms were not due to the direct physiological effects of a substance(e.g., a drug of abuse, a medication) or a general medical condition (e.g., hypothyroidism) and the symptoms caused clinically significant impairment in social, occupational, or other important areas of functioning.

#### Substance use, chronic illness and medication use

With regard to substance use, students were asked if they ever smoked tobacco, drank alcohol, or took other drugs (such as marijuana, cocaine, inhalants, and hallucinogens) in a 4-week period prior to the interview. With regard to chronic physical illness, students were provided with a list of chronic conditions (e.g. HIV/AIDS, diabetes, asthma and hypertension) and asked to indicate whether or not they had experienced an episode of any those conditions in a 4-week period prior to the interview. With regard to chronic medication use, students were asked if they were required to take medications for the chronic medical condition that they had.

### Statistical analyses

Statistical analysis was carried out with SPSS, version 11.5. Frequencies of participants’ characteristics were computed and logistic regression analyses conducted to determine associations between various participant characteristics and significant depression symptoms. For the bivariate analyses, we used Chi square tests or Fisher’s exact test for categorical variables, and independent-sample t tests for continuous variables. Factors that had a significant bivariate association (p ≤ 0.05) with depression symptoms were then included in a multi-variate logistic regression model. We assessed for multicollinearity by computing the variance inflation factor for the variables in the model.

## Results

### Sample characteristics

Of the 541 study participants that we approached to take part in the study, 519 (96 %) completed the study questionnaires. The majority were males 301 (58 %), and 306 (59 %) were in the age range of 14–16 years with a mean age of 16 years (SD 2.18). A total of 155 (30 %) participants were orphans. Detailed baseline characteristics of the study participants are presented in Table 1.

**Table 1:** Frequency of demographic characteristics of the adolescents (N = 519)

Variable	n	%
Gender		
Male	301	58.0
Female	218	42.0
Age (years)		
<14	47	9.0
≥14	472	91
Type of school		
Boys only (boarding)	163	31.4
Girls only (boarding)	80	15.4
Boarding mixed	118	22.7
Day mixed	158	30.4
Nature of family		
Single parent	108	20.8
Polygamous	155	29.9
Monogamous	256	49.3
Head of household		
Father	342	65.9
Mother	122	23.5
Child	10	1.9
Other	45	8.7
Orphan hood		
Not orphan	364	70.1
Paternal orphan	84	16.2
Maternal orphan	34	6.6
Double orphan	37	7.1

### Prevalence and factors associated with depression symptoms

Of 519 participants screened with the CDI, 109 (21 %) had significant depression symptoms. Of the 109 participants with significant depression symptoms, only 74 were evaluated with the MINI-KID (Table 2) and of these, 8 (11 %) met criteria for major depression and 6 (8 %) met criteria for dysthymia. Therefore, among participants that were assessed with both the CDI and the MINI-KID (n = 484), the prevalence of depressive disorders was 2.9 %. In this sample, 15 (3.1 %) reported current suicidal ideation. Table 3 illustrates the results of the bivariate logistic regression analyses. Results from multivariate analysis indicate that gender (adjusted odds ratio [AOR] 1.50, 95 % CI 1.01–2.01, p ≤ 0.05), living in child headed household (AOR 2.20, 95 % CI 1.11–3.62, p ≤ 0.05), chronic physical illness (AOR 1.25, 95 % CI 1.10–3.02, p ≤ 0.05) and orphan hood (AOR 1.20, 95 % CI 1.00–2.02, p ≤ 0.05) were each independently associated with significant depression symptoms. All variables in the model had a variance inflation factor less than 5 indicating that multicollinearity was not of concern in this model. The commonest psychiatric disorders found among those with significant depression symptoms were social phobia (30 %), panic disorder with or without agoraphobia (28 %), specific phobia (26 %), separation anxiety (16 %), obsessive–compulsive disorder (15 %), conduct disorder (11 %) and alcohol dependence disorder (3 %).

**Table 2:** Current MINI KID psychiatric disorder amongst the students with CDI scores ≥ 19

DSM IV diagnosis	Frequency	Percentage N = 74	Percentage of total population N = 519
Major depression	8	10.8	1.5
Dysthymia	6	8.1	1.2
Panic disorder with agora phobia (current)	3	4.15	0.6
Panic disorder without agoraphobia (current)	18	24.3	3.5
Separation anxiety (current)	12	16.21	2.3
Social phobia (current)	22	29.72	4.2
Specific phobia (current)	19	25.67	3.7
Obsessive compulsive disorders (current)	11	14.86	2.1
Post-traumatic stress disorder (current)	4	5.4	0.8
Alcohol dependence (current)	2	2.7	0.4
Conduct disorder	8	10.8	1.5

**Table 3:** Comparison of demographic, family and social characteristics of the adolescents by CDI scores for depression

Variable	Study sample (N = 519)	Depression CDI ≥ 19	No depression CDI < 19	OR (95 % CI)	*P* value
Gender					
Male	301	52 (17)	249 (83)	1	
Female	218	57 (26)	161 (74)	1.7 (1.1–2.6)	*0.01*
Age					
<14	47	9 (19)	38 (81)	1	
≥14	472	100 (21)	372 (79)	0.99 (0.63–1.55)	0.95
Type of school					
Boys only	163	20 (12)	143 (88)	1	
Girls only	80	26 (33)	54 (68)	2.07 (1.18–3.60)	*0.01*
Boarding mixed	118	29 (25)	89 (75)	1.31 (0.78–2.18)	0.27
Day mixed	158	34 (22)	124 (78)	1.06 (0.65–1.71)	0.81
Nature of family					
Monogamous	256	43 (17)	213 (83)	1	
Polygamous	155	33 (21)	122 (79)	1.03 (0.63–1.66)	0.91
Single parent	108	33 (31)	75 (69)	1.94 (1.71–3.22)	*0.01*
Head of household					
Adult	509	104 (20)	405 (80)	1	
Child	10	5 (50)	5 (50)	3.85 (1.07–16.70)	*0.02*
Orphan hood					
Not orphaned	364	59 (16)	305 (84)	1	
Orphaned	155	50 (32)	105 (68)	1.4 (1.61–3.84)	*0.05*
Chronic physical illness					
Absent	381	73 (19)	308 (81)	1	
Present	138	36 (26)	102 (74)	1.64 (1.03–2.63)	*0.03*
Medication					
Absent	381	79 (21)	302 (79)	1	
Present	138	30 (22)	108 (78)	0.91 (0.61–0.97)	0.97
Alcohol/substance use					
Absent	471	94 (20)	377 (80)	1	
Present	48	15 (31)	33 (69)	1.76 (0.9–3.4)	0.08

## Discussion

This study contributes to the research literature on prevalence and factors associated with depression symptoms among school-going adolescents in sub-Saharan Africa. The prevalence estimate of depression symptoms in this study of 21 % is high and is likely to impair the adolescents’ ability to achieve academically and other areas of functioning. The prevalence of 21 % falls within the range of prevalence estimates obtained from previous studies conducted in both developing [14, 19] and developed countries that used depression screening instruments [33–35]. Likewise the prevalence rate of depressive disorder of 2.9 % that we found in this study is similar to what has been reported in studies conducted in the United States where a formal diagnosis of depression has been made among study samples of adolescents [36]. In this study, Kessler and colleagues analyzed data from 10,123 school-going adolescents in the age range of 13–17 years and found a prevalence rate of depressive disorder of 2.6 %. The high rates of depressive symptoms may also be due to general psychosocial distress resulting from general hardships in living, school related stress and poverty while the low rates of Major depressive disorder could be explained by the factors that promote resilience. In our study the research participants were secondary school students, and some of them could have come from high social economic class which has been found to be protective against depressive illness. Indeed Klassen et al. in their study on resilience in former Ugandan child soldiers, found that 27.6 % showed posttraumatic resilience as indicated by the absence of posttraumatic stress disorder, depression as well as clinically significant behavioural and emotional problems. This was attributed to better socioeconomic situation in the family, and more perceived spiritual support among other factors [37]. On the other hand, one would think that the low rates of depression (as measured by MINI KID) could have been a consequence of the selection bias as 35 students out of 109 students who had scored ≥19 points on the CDI were not interviewed. However these students may have left school for other reasons such as poverty, peer influence (Table 2).

In keeping with findings from previous studies, the prevalence of depressive symptoms was more than twice as common in girls as in boys. The excess of affected girls is seen in epidemiological as well as clinical samples, and is robust across different methods of assessment. Previous researchers have explained that sex differences in rates of depression are therefore unlikely to be merely due to differences in help-seeking or reporting of symptoms [38]. Although the reasons for this post-pubertal-onset sex difference are not fully understood, recent studies indicate that this difference is probably due to some combination of age-related changes in biological or social circumstances [39, 40].

The significant association between psychosocial stressors such as being a double orphan, living in a child headed household, and the presence of significant depressive symptoms is not surprising as such stressors have been reported to be significantly associated with adolescent depression and suicidality [41]. In South Africa, Cluver and colleagues found that acquired immuno-deficiency syndrome (AIDS)—orphaned children showed higher depression, anxiety, and post-traumatic stress disorder (PTSD) scores when compared with other-orphans and non-orphans [42]. El-Missiry and colleagues, studied depression in adolescent girls in Egypt using the CDI and found that presence of significant depression symptoms was associated with psychosocial stressors such as, quarrelsome family atmosphere, socioeconomic status, and negative life events [19].

The association between alcohol and drug use and the presence of depressive symptoms in this study is consistent with findings from previous studies [43, 44]. We noted a trend towards greater likelihood of alcohol and drug use in participants with significant depression symptoms than in those without. However, as our data are of a cross-sectional nature, it is not possible to make any inferences about whether the depression symptoms led to alcohol use or vice versa. Thus, longitudinal studies are needed to address this issue.

Consistent with findings from a systematic review of 340 studies investigating the relationship between depressive symptoms in children and adolescents with chronic physical illness [45], the adolescents who reported the presence of a chronic physical illness were more likely to have significant depression symptoms than those who did not report such an illness. Previous researchers have explained that the myriad of complex challenges associated with chronic disease conditions may interfere with regular school attendance [46–48], lead to peer rejection which may have detrimental effects on their self-concept [49, 50] and may result in inappropriate parental attitudes and behaviors, which may impair psychological well-being [51].

This study has limitations. First, as the study sample consisted of school-going adolescent in one district we cannot generalize our findings other districts elsewhere in Uganda or other sub-Saharan developing countries. Second, this study did not assess for parental factors and other factors such as coping styles or social support all of which have been associated with adolescent depression in previous studies. Third, the absence of collateral information may maximize effects of recall bias. Fourth, information was collected on exposures and outcomes simultaneously, thus causal relationships are difficult to establish. Fifth, the study did not include those who left school for a variety of reasons yet those who left school could have done so for reasons of depression. Indeed 35 students out of 109 students who had scored ≥19 points on the CDI were not interviewed with the MINI KID as they had left school and this could have affected the prevalence rates. Consequently, this study will only give clues as to whether certain factors may or may not be potential etiological factors of depression symptoms in school-going adolescents in central Uganda. Therefore, studies with better epidemiological design such as the case–control study can be used to investigate risk factor for depression in school-going adolescents.

Despite these limitations, this study, to our knowledge, provides the first prevalence estimates of depression symptoms among a sample of school going-adolescents in a non-conflict region in Uganda. Our study has important implications for school health programs in particular the integration of mental health issues into the school health education and health services. First, school health programs need to embrace locally adapted simple tools to measure depression which will enable us to distinguish depressive symptoms from clinical syndromes of depression because management strategies are different. Second, there is a need to offer stress management programs in which stressful situations among adolescent can be addressed before they affect emotional well-being, this research provides an important first step into current understanding of depression among school-going adolescents, which could be useful in designing school interventions for depression. Thirdly, mental health education for all stakeholders in the education sector must be scaled up to enhance early diagnosis and early interventions.

## Conclusion

Significant depression symptoms are highly prevalent among this sample of school-going adolescents and may progress to full-blown depressive disorders. Integration of culturally sensitive psychological interventions to prevent and treat depression among school-going adolescents is desperately needed. There is great need for a child and adolescent mental health policy that will be used to plan for mental health services in schools.

## Abbreviations

AIDS: acquired immuno-deficiency syndrome
CDI: Children Depression Inventory
DSM IV: Diagnostic and Statistical Manual of Mental Disorders, 4th Edition
DALYs: disability-adjusted life years
HIV: human immune deficiency virus
MINI: KID mini international neuro-psychiatric interview for children and adolescents
PTSD: post-traumatic stress disorder
